# Novel approach for the detection of tubular cell migration into the interstitium during renal fibrosis in rats

**DOI:** 10.1186/s13069-015-0030-0

**Published:** 2015-07-10

**Authors:** Masao Nakasatomi, Akito Maeshima, Keiichiro Mishima, Hidekazu Ikeuchi, Toru Sakairi, Yoriaki Kaneko, Keiju Hiromura, Yoshihisa Nojima

**Affiliations:** Department of Medicine and Clinical Science, Gunma University Graduate School of Medicine, 3-39-15 Showa, Maebashi, 371-8511 Japan

## Abstract

**Background:**

The process of epithelial-mesenchymal transition (EMT), which is generally defined by phenotypic changes of injured tubules such as loss of epithelial markers or acquisition of mesenchymal markers, implies various activating steps, including proliferation, migration, and ability to produce extracellular matrix proteins. We established here a novel approach for the detection of tubular cell migration into the interstitium during renal fibrosis in vivo.

**Results:**

Using an osmotic pump, bromodeoxyuridine (BrdU) was continuously given to 7-week-old Wistar rats for 4 weeks, and BrdU-positive cells were detected by immunostaining. BrdU-positive cells were present in aquaporin-1-positive proximal tubules, but not in the interstitium of BrdU-treated rat kidneys. After unilateral ureteral obstruction (UUO), some BrdU-positive tubular cells protruded from the basement membrane and migrated into the interstitium. Interstitial BrdU-positive cells were co-localized with alpha-smooth muscle actin, fibroblast specific protein-1, vimentin, and type I collagen, but not with CD68 or CD3. No BrdU-positive cells were observed in the interstitium of sham-operated kidneys. The number of BrdU-positive cells migrating into the interstitium significantly increased and peaked at 8 days after UUO.

**Conclusions:**

Long-term BrdU labeling marked some of the proximal tubular cells and enabled us to detect tubular cell migration into the interstitium after UUO. This simple method might be useful to detect EMT in vivo.

## Background

Epithelial-mesenchymal transition (EMT) is one of the critical processes involved in renal tubulointerstitial fibrosis, which is a common pathway in various kidney diseases, leading to functional deterioration and eventual loss of renal function, regardless of initial cause [1, 2]. As the disease progresses, tubular epithelial cells lose their apical-basal polarity, become elongated, and migrate into the peritubular interstitium via the disrupted tubular basement membrane. During this process, damaged tubular cells lose the epithelial cell marker E-cadherin and acquire mesenchymal features such as α-smooth muscle actin (α-SMA) or vimentin and produce interstitial matrix components such as fibronectin and type I collagen [3, 4].

Increasing evidence from animal models with renal injury or human data using renal biopsy samples indicates that EMT plays a decisive role in the evolution of renal tubulointerstitial fibrosis [5]. It has been shown that proximal tubular epithelial cells can undergo EMT in vitro in response to TGF-β1 [6]. In human kidney diseases, tubular epithelial cells, through their transition to a mesenchymal phenotype, produce extracellular matrix proteins and directly intervene in fibrotic processes [7]. It has been also reported that early phenotypic changes indicative of EMT predict the progression toward interstitial fibrosis in human renal allografts [8–10]. In 5/6-nephrectomized rats, the injured tubular epithelial cells become positive for α-SMA, a marker of myofibroblasts. Tubular labeling by α-SMA increases with time after nephrectomy and is correlated with tubular basement membrane disruption, and signs of possible tubular cell migration toward the interstitium [11]. The pathogenetic role of EMT has also been demonstrated by in vivo observations using transgenic reporter mice in which fibroblasts were specifically labeled with green fluorescent protein. Taking this approach, it was demonstrated that interstitial fibroblasts are partially derived from local EMT during renal fibrogenesis [12].

The EMT process is defined at present by the observation that cells of injured tubules lose epithelial markers while they acquire mesenchymal marker proteins. Structural details of this process have not yet been elucidated by either transmission electron microscopy or light microscopy. In the present study, we developed a simple method for detection of tubular cell movement into the interstitium in vivo using long-term bromodeoxyuridine (BrdU) labeling. This classical technique marks proximal tubular nuclei with BrdU and enabled us to perform quantitative assessment of tubular cell migration in vivo. Given that EMT in the kidney is of significant interest as a therapeutic target [13], this method may be applicable to the screening of new drugs targeting EMT.

## Results

### Presence of BrdU-positive cells in a normal rat kidney after long-term BrdU labeling

**Fig. 1:**
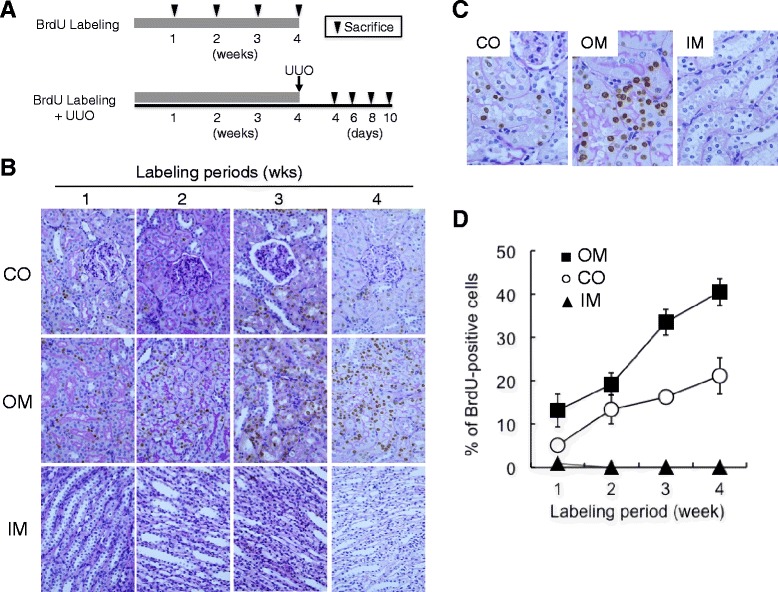
Presence of BrdU-positive cells in the normal rat kidneys after long-term BrdU labeling. **a** Experimental design. BrdU was intraperitoneally injected into normal rats for the indicated periods. After BrdU labeling, the rats were sacrificed, and the kidneys were removed and used for histological examination. In another experiment, UUO was induced in rats pre-treated with BrdU for 4 weeks as described in “Methods”. At the indicated times after UUO, the rats were sacrificed and the kidneys were removed and used for histological examination. **b**, **c** Detection of BrdU-positive cells in the normal rat kidneys after long-term BrdU labeling. BrdU was injected intraperitoneally into the normal rats for the indicated periods. BrdU-positive cells (brown nuclei) were examined by immunostaining and were counterstained with PAS. Magnification is ×400 in **b** and ×1000 in **c**. *CO* cortex, *OM* outer medulla, *IM* inner medulla. **d** Quantitative analysis of BrdU-positive cells. (*Filled square*) OM, outer medulla, (*empty circle*) CO, cortex, (*filled triangle*) IM, inner medulla. Values are means ± SE (*n* = 5)

BrdU is a thymidine analog useful for the detection of cells undergoing DNA synthesis. We previously demonstrated that there is a small population of renal tubular cells in the normal rat kidney that retain BrdU in their nuclei after 1 week of BrdU labeling, followed by a 2-week chase period [14–16]. These findings raise the possibility that continuous long-term labeling with BrdU is an alternative method for marking renal tubular cells. To test this idea, BrdU was continuously given to 7-week-old Wistar rats using an osmotic pump for various periods and localization of BrdU-positive cells was examined by immunostaining (Fig. 1a). Some BrdU-positive cells were present in renal tubules of the cortex and the outer medulla of the kidney after a 1-week BrdU labeling. After 4 weeks of BrdU labeling, numerous BrdU-positive cells were detected in the cortex and outer medulla, but not the inner medulla of the normal rat kidney (Fig. 1b, c). Quantitative analysis showed that the number of BrdU-positive cells was positively associated with labeling period (Fig. 1d).

### Localization of BrdU-positive cells in a normal rat kidney after long-term BrdU labeling

**Fig. 2:**
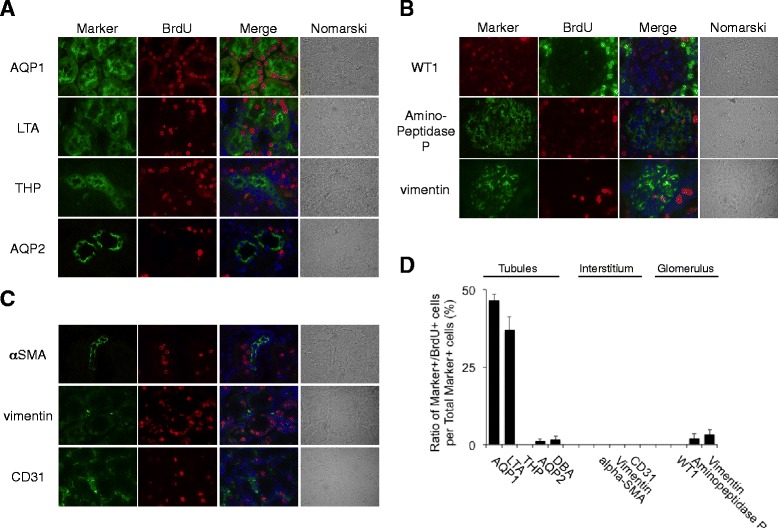
Localization of BrdU-positive cells in the normal rat kidneys after long-term BrdU labeling. **a–c** BrdU was given intraperitoneally to normal rats for 4 weeks. Double staining of BrdU with several tubular (**a**), glomerular (**b**), and interstitial (**c**) markers was performed. Markers: aquaporin-1 (*AQP-1*), lotus tetragonolobus agglutinin (*LTA*), Tamm-Horsfall glycoprotein (*THP*), aquaporin-2 (*AQP-2*), *alpha-SMA*, *vimentin*, *CD31*, *WT1*, and *aminopeptidase P*. DAPI (*blue*). Magnification, ×1000. **d** Quantitative analysis of percentage of BrdU-positive cells per total marker-positive cells. Values are means ± SE (*n* = 5)

Using several nephron markers, we also examined the localization of BrdU-positive cells in the kidney after long-term BrdU labeling. Most BrdU-positive cells were present in aquaporin (AQP)-1 or lotus tetragonolobus agglutinin (LTA)-positive proximal tubules, but not in other nephron segments (Fig. 2a). A very small number of BrdU-positive cells were detected in the glomerulus and were co-localized with aminopeptidase P-positive endothelial cells and vimentin-positive mesangial cells, but not with WT1-positive podocytes (Fig. 2b). Few BrdU-positive cells were observed in the interstitium of the normal rat kidney. No BrdU-positive cells were co-localized with vimentin-positive fibroblasts, CD31-positive capillary endothelial cells, or α-SMA-positive smooth muscle cells of vessels (Fig. 2c). Quantitative analysis demonstrated that nearly all BrdU-positive cells were proximal tubules (Fig. 2d).

### Migration of BrdU-positive tubular cells into interstitium after UUO

We next examined the localization of BrdU-positive cells in the kidney of 4-week BrdU-treated rats after unilateral ureteral obstruction (UUO). UUO was induced in these BrdU-treated rats, and the kidneys were removed for analysis at 4, 6, 8, and 10 days after UUO (Fig. 1a).

**Fig. 3:**
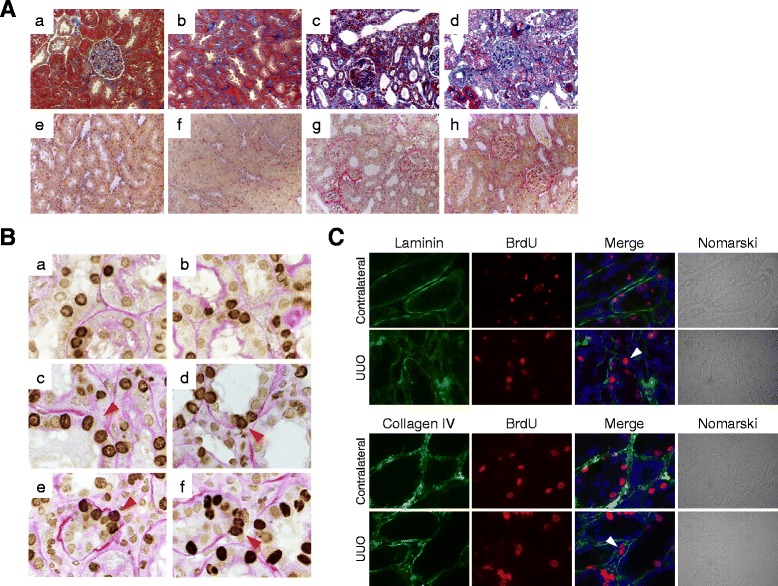
Migration of BrdU-positive tubular cells into the interstitium of the UUO kidneys. **a** Masson’s trichrome staining (*a*–*d*) and EVG staining (*e*–*f*) of kidney sections. (*a*, *e*) Normal kidney; (*b*, *f*) contralateral kidney, 10 days; (*c*, *g*) UUO kidney, 6 days; and (*d*, *h*) UUO kidney, 10 days. Magnification, ×200. **b** Localization of BrdU-positive cells in the normal (*a*), contralateral (*b*), and UUO (*c*–*f*) kidneys of rats with long-term BrdU labeling. Magnification, ×1000. *Arrowheads* indicate BrdU-positive cells protruding from TBM or migrating into interstitium. **c** Destruction of tubular basement membrane in the UUO kidneys of rats with long-term BrdU labeling. *BrdU* (*red*), markers (*green*), and DAPI (*blue*). Magnification, ×1000

Masson’s trichrome staining (Fig. 3a (*a*–*d*)) as well as elastin-van Gieson (EVG) staining (Fig. 3a (*e*–*h*)) demonstrated the deposition of extracellular matrix in the UUO kidneys at 6 days (Fig. 3a (*c*, *g*)) or at 10 days (Fig. 3a (*d*, *h*)) after operation, but not in the normal kidneys (Fig. 3a (*a*, *e*)) or contralateral kidneys (Fig. 3a (*b*, *f*)), demonstrating the establishment of renal fibrosis in this rat UUO model.

In normal (Fig. 3b (*a*)) or sham-operated (Fig. 3b (*b*)) kidneys, BrdU-positive signals were observed in the nuclei of renal tubular cells. After UUO, part of the tubular basement membrane adjacent to BrdU-positive cells was disrupted (Fig. 3b (*c*)). Some BrdU-positive tubular cells protruded from the destroyed basement membrane (Fig. 3b (*d*, *e*)) and were found in the interstitium of the UUO kidneys (Fig. 3b (*f*)). Few BrdU-positive cells were observed in the interstitium of the sham-operated kidneys (data not shown).

We next performed immunostaining for laminin and type IV collagen, the components of tubular basement membrane (Fig. 3c). In the contralateral or sham-operated kidneys (data not shown), the tubular basement membrane was preserved throughout the experiments. In contrast, loss of laminin or type IV collagen expression was observed in some kidney tubules after UUO. Some BrdU-positive cells were adjacent to the destroyed tubular basement membrane or were localized in the interstitium of the UUO kidneys (Fig. 3c).

### Phenotypic changes in BrdU-positive cells after UUO

**Fig. 4:**
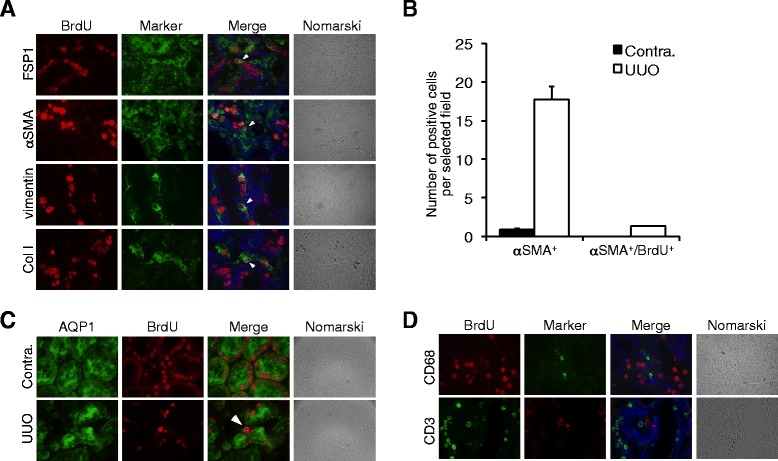
Phenotypes of BrdU-positive tubular cells in the UUO kidneys. **a** Expression of myofibroblast-related markers in kidneys at 7 days after UUO. *BrdU* (*red*), *markers* (*green*), and DAPI (*blue*). Magnification, ×1000. *Arrowheads* indicate BrdU-positive cells coexpressing myofibroblast markers. **b** Number of *alpha-SMA-positive* cells and *alpha-SMA/BrdU double-positive* cells in the interstitium of contralateral and UUO kidneys at 8 days after operation. Values are means ± SE (*n* = 5). **c** Expression of *AQP-1* in kidneys at 7 days after UUO. *Arrowheads* indicate BrdU-positive cells losing AQP-1 expression. *BrdU* (*red*), *AQP-1* (*green*), and DAPI (*blue*). Magnification, ×1000. **d** Expression of inflammatory cell markers in kidneys at 7 days after UUO. DAPI (*blue*). Magnification, ×1000

We further examined the phenotype of BrdU-positive cells using several mesenchymal or epithelial markers. No BrdU-positive cells expressed mesenchymal markers such as fibroblast specific protein (FSP)-1, α-SMA, and vimentin in the normal, contralateral, and sham-operated kidneys (data not shown). After UUO, some BrdU-positive cells became positive for FSP-1, α-SMA, and vimentin (Fig. 4a), and some BrdU-positive cells produced type I collagen in the UUO kidneys (Fig. 4a). Quantitative analysis showed that α-SMA-positive cells were abundantly present in the interstitium of the UUO kidneys but not of the contralateral kidneys (Fig. 4b). The total count of α-SMA/BrdU double-positive cells corresponds to approximately 7 % of the total α-SMA-positive cells (Fig. 4b). On the other hand, some BrdU-positive tubular cells, which were AQP-1-positive in normal or sham-operated kidneys (data not shown), lost AQP-1 expression after UUO (Fig. 4c). These data suggest that some BrdU-positive tubular cells lose epithelial phenotype but acquire mesenchymal phenotype, during renal fibrosis.

Infiltration of inflammatory cells such as CD68-positive macrophages or CD3-positive T lymphocytes were observed in the interstitium of the UUO kidneys but were not co-localized with BrdU-positive cells (Fig. 4d).

### Increases in number of BrdU-positive cells migrating into the interstitium after UUO

**Fig. 5:**
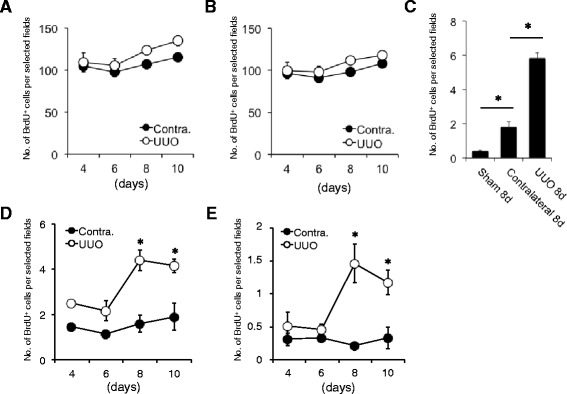
Number of BrdU-positive tubular cells migrating into the interstitium of the UUO kidneys. **a** Total count of BrdU-positive cells in the contralateral and UUO kidneys at 4, 6, 8, and 10 days after operation. Values are means ± SE (*n* = 5). **b** Number of BrdU-positive tubular cells in contralateral and UUO kidneys at 4, 6, 8, and 10 days after operation. Values are means ± SE (*n* = 5). **c** Total count of interstitial BrdU-positive cells and BrdU-positive cells on crossing TBM in the sham-operated, contralateral, and UUO kidneys at 8 days after operation. Values are means ± SE (*n* = 5). *P < 0.05 vs. sham-operated kidneys or contralateral kidneys. **d** Number of BrdU-positive cells on crossing TBM in the contralateral and UUO kidneys at 4, 6, 8, and 10 days after operation. Values are means ± SE (*n* = 5). *P < 0.05 vs. contralateral kidneys on the same days. **e** Number of interstitial BrdU-positive cells in the contralateral and UUO kidneys at 4, 6, 8, and 10 days after operation. Values are means ± SE (n = 5). *P < 0.05 vs. contralateral kidneys on the same days

We finally performed quantitative analysis of BrdU-positive cells migrating into the interstitium of the UUO kidneys. The total count of BrdU-positive cells (Fig. 5a) as well as BrdU-positive tubular cells (Fig. 5b) in the kidneys of rats with a 4-week BrdU label gradually increased after UUO. Interstitial BrdU-positive cells and BrdU-positive cells on crossing tubular basement membrane (TBM) were almost undetectable in sham-operated kidneys but were slightly detectable in contralateral kidneys at 8 days after UUO. The total number of interstitial BrdU-positive cells and BrdU-positive cells on crossing TBM in the UUO kidneys was significantly higher than that of the contralateral kidneys (Fig. 5c). The number of BrdU-positive cells on crossing TBM peaked at 8 days after UUO (Fig. 5d). The interstitial BrdU-positive cell number also increased after UUO and reached a maximum at 8 days after operation (Fig. 5e).

## Discussion

Generally, the process of EMT implies various activating steps, including proliferation, phenotype changes, migration, and ability to produce extracellular matrix proteins [13]. The origin of interstitial fibroblasts in the fibrotic kidneys is considered to be heterogeneous in mice [3]. They are composed of resident fibroblasts, bone marrow-derived cells, and interstitial cells via endothelial mesenchymal transition (EndMT) or EMT [17]. Using gene-targeting techniques, previous studies have elegantly demonstrated that renal epithelial tubules labeled with markers such as LacZ or GFP are present in the interstitium after induction of renal fibrosis, thus suggesting that renal tubular cells transdifferentiate into myofibroblasts. A small number of cells positive for FSP-1, a marker for renal interstitial fibroblasts [18, 19], arise via local EMT, express collagen type I, and expand by cell division during tissue fibrosis [12, 20]. Using multiple genetically engineered mice, recent reports also demonstrated that the origin of α-SMA-positive myofibroblasts is heterogeneous and that 5 % of these originated from the EMT [21]. In contrast, several studies have raised doubts about the existence of EMT in vivo. These studies have fate-traced epithelial cells in three different mouse models of kidney disease and failed to detect myofibroblasts originating from epithelium, instead demonstrating the pericyte origin of myofibroblasts [22, 23]. The degree of EMT contribution to renal fibrosis may differ depending on the myofibroblast markers used or genetic variations in mouse strains in susceptibility to renal injury [20, 24, 25]. It remains unknown whether this concept regarding heterogeneity of interstitial fibroblasts is applicable to “rat” or “human” kidneys.

Our method has several limitations. Long-term BrdU labeling marks some, but not all, proximal tubular cells. Therefore, migration of BrdU-negative proximal tubular cells or other parts of nephron segments into the interstitium cannot be detected in this way. Cell division also affects the sensitivity of BrdU detection. Incorporated BrdU label would also be diluted out and become undetectable after multiple cell divisions, making it difficult to trace all descendants from BrdU-positive cells, if they are actively dividing. It has been reported that high concentrations of BrdU are toxic for cell survival and reduce cell viability, raising the possibility that incorporation of BrdU in chromosomes may affect cell phenotype or increase the probability of EMT during renal fibrosis. It has been also reported that interstitial fibroblasts after UUO are partially derived from the bone marrow [12, 21]. In this study, we found that many BrdU-positive cells are localized in the bone marrow of the rats with a 4-week BrdU label. There are no significant differences in the number of BrdU-positive bone marrow cells between the sham-operated rats and UUO rats (unpublished observation). No BrdU-positive cells were co-localized with CD68-positive cells or CD3-positive cells in the interstitium of the UUO kidneys (Fig. 4d); however, it is still possible that bone marrow-derived BrdU-positive cells migrate into the interstitium after UUO.

It has been shown that certain soluble factor(s) produced by the UUO kidneys affect the contralateral kidneys. For example, moderate medullary TGF-beta immunoreactivity is observed in the contralateral kidneys, but not in the sham-operated kidneys in a rat UUO model [26]. Consistent with this observation, we detected a small number of interstitial BrdU-positive cells in the contralateral kidneys, but not in the sham-operated kidneys (Fig. 5c), thus suggesting the presence of certain circulating factor(s) that can induce tubular cell migration into the interstitium.

A key question remains about the turnover of proximal tubular cells under normal conditions. In the present study, proximal tubular cells are marked with BrdU after long-term BrdU labeling, probably because these cells have a slow-cycling nature. However, it remains unknown which external or internal cues control proximal tubular cell cycles. The differences in structural architecture between BrdU-positive and BrdU-negative cells also remain uncertain. Further study, such as immunoelectron microscopic analysis, will be necessary to clarify this issue.

## Conclusions

In the present study, we established a simple alternative method for detection of tubular cell movement during renal fibrosis. We found that long-term in vivo BrdU labeling is able to mark some of the proximal tubular cells and that some BrdU-positive cells lose epithelial marker expression, migrate into the interstitium, and acquire mesenchymal marker expression after UUO. Incorporated BrdU remains in the nucleus of proximal tubular cells, even after these cells migrate into the interstitium and change their phenotype. This enabled us to trace their movement during renal fibrosis and count their cell number. This simple non-gene targeting approach would be useful to detect EMT in vivo.

## Methods

### In vivo BrdU labeling

Male Wistar rats weighing 200 g each were obtained from Charles River Japan (Tokyo, Japan). Using ALZET osmotic pumps (DURECT, Cupertino, CA), BrdU (20 mg/kg/day), an analog of thymidine, was intraperitoneally administered to the rats for the indicated periods. After BrdU labeling, the rats were sacrificed, and the kidneys were removed, fixed with 10 % formaldehyde, and embedded in paraffin. Sections (4 μm) were immunostained using mouse anti-BrdU antibody (GE Healthcare, Buckinghamshire, UK) or rat anti-BrdU antibody (AbD Serotec, Oxford, UK) according to the manufacturer’s instructions and were counterstained with periodic acid-Schiff.

### Unilateral ureteral obstruction

UUO was performed as described previously [27]. Briefly, after induction of general anesthesia by intraperitoneal injection of pentobarbital (50 mg/kg body wt), the abdominal cavity was exposed via a midline incision and the left ureter was ligated at two points with 2–0 silk. At the indicated times after UUO, the rats were sacrificed and the kidneys were removed and used for histological examination. Ureteral obstruction was confirmed by observation of pelvic dilatation and proximal ureter and collapse of the distal ureter. Sham surgery was performed in a similar manner, except without ligating the left ureter. Experimental protocols were approved by the Ethics Review Committee for Animal Experimentation of Gunma University.

### Quantification of BrdU-positive cells undergoing EMT

Quantitative analysis of BrdU-positive cells was performed by counting the positive nuclei from ten randomly selected fields under a light microscope at ×400 magnification. BrdU-positive tubular cells protruding from the basement membrane and BrdU-positive cells localized in the interstitium were defined as tubular cells undergoing EMT. Desquamated BrdU-positive cells in the lumen were excluded from the analysis. The average of the ten counts was calculated. The number of BrdU-positive cells was quantified and expressed as a percentage of total cells per field. Data are presented as means ± SE (*n* = 5).

### Indirect fluorescence immunohistochemistry

Immunostaining was performed as described previously [28]. The paraffin-embedded sections (4 μm) were deparaffinized, rehydrated in a routine manner, and incubated at 4 °C overnight with primary antibodies. After washing with phosphate buffered saline (PBS), the sections were incubated with fluorescein-labeled secondary antibodies and 4′-diamidino-2-phenylindole (DAPI: Boehringer Mannheim) at room temperature (RT) for 1 h.

Frozen sections (6 μm) embedded with Tissue-Tek OCT compound (Miles, Inc., Elkhart, IN) were also used. The sections were fixed with cold methanol mixed with equivalent amounts of acetone for 15 min at −20 °C and autoclaved at 120 °C for 10 min in 10 mmol/citric acid buffer to retrieve antigens. Immunofluorescent images were recorded, as described previously [27]. Antibodies used in this study were as follows: rabbit polyclonal anti-laminin antibody; rabbit anti-mouse type IV collagen antibody (Progen Biotechnik GmbH, Heidelberg, Germany); Fluorescein-labeled Lotus Tetragonolobus Lectin (Vector, Burlingame, CA); goat anti-Tamm-Horsfall glycoprotein (THP) antibody (Biomedical Technologies, Inc., Ward Hill, MA); goat anti-aquaporin (AQP)-2 antibody (BioVision Research Products, Milpitas, CA); mouse monoclonal anti-α-smooth muscle actin (SMA) antibody (Sigma Aldrich, St. Louis, MO); rabbit anti-vimentin antibody (Cell Signaling Technology, Danvers, CO); mouse anti-rat endothelial aminopeptidase P antibody (eBioscience, Vienna, Austria); rabbit anti-rat type I collagen antibody (LSL, Tokyo, Japan); goat anti-human S100A4 (FSP-1) antibody (Acris Antibodies, Herford, Germany); mouse anti-rat CD68 antibody (AbD Serotec, Oxford, UK); rabbit anti-AQP-1 antibody; goat anti-CD31 antibody, mouse anti-CD3 antibody (Santa Cruz Biotechnology, Dallas, TX); and rabbit anti-Wilms Tumor Protein antibody (Abcam, Cambridge, MA).

### Histological examination

The paraffin-embedded sections (4 μm) were deparaffinized and rehydrated in a routine manner, and renal fibrotic changes were assessed by Masson’s trichrome staining and EVG staining.

### Statistical analysis

Differences between means were compared by Student’s *t* test, with *P* values of <0.05 considered to be significant.

## Abbreviations

α-SMA: α-smooth muscle actin
AQP-1: aquaporin-1
AQP-2: aquaporin-2
BrdU: bromodeoxyuridine
EMT: epithelial-mesenchymal transition
FSP-1: fibroblast specific protein-1
LTA: lotus tetragonolobus agglutinin
UUO: unilateral ureteral obstruction
